# A Review on Machining SiCp/Al Composite Materials

**DOI:** 10.3390/mi15010107

**Published:** 2024-01-07

**Authors:** Zhigao Chen, Fei Ding, Zhichao Zhang, Qiuyan Liao, Zheng Qiao, Yuan Jin, Mingjun Chen, Bo Wang

**Affiliations:** Center for Precision Engineering, Harbin Institute of Technology, Harbin 150001, China; zhigaochen01@outlook.com (Z.C.); fei.ding@hit.edu.cn (F.D.); 22s008130@stu.hit.edu.cn (Z.Z.); 19b908146@stu.hit.edu.cn (Q.L.); qiaozheng@hit.edu.cn (Z.Q.); jinyuan@hit.edu.cn (Y.J.); chenmj@hit.edu.cn (M.C.)

**Keywords:** SiCp/Al composite materials, machining, mechanism, tool wear, machinability

## Abstract

SiCp/Al composite materials are widely used in various industries such as the aerospace and the electronics industries, primarily due to their excellent material properties. However, their machinability is significantly weakened due to their unique characteristics. Consequently, efficient and precise machining technology for SiCp/Al composite materials has become a crucial research area. By conducting a comprehensive analysis of the relevant research literature from both domestic and international sources, this study examines the processing mechanism, as well as the turning, milling, drilling, grinding, special machining, and hybrid machining characteristics, of SiCp/Al composite materials. Moreover, it summarizes the latest research progress in composite material processing while identifying the existing problems and shortcomings in this area. The aim of this review is to enhance the machinability of SiCp/Al composite materials and promote high-quality and efficient processing methods.

## 1. Introduction

**Figure 1:**
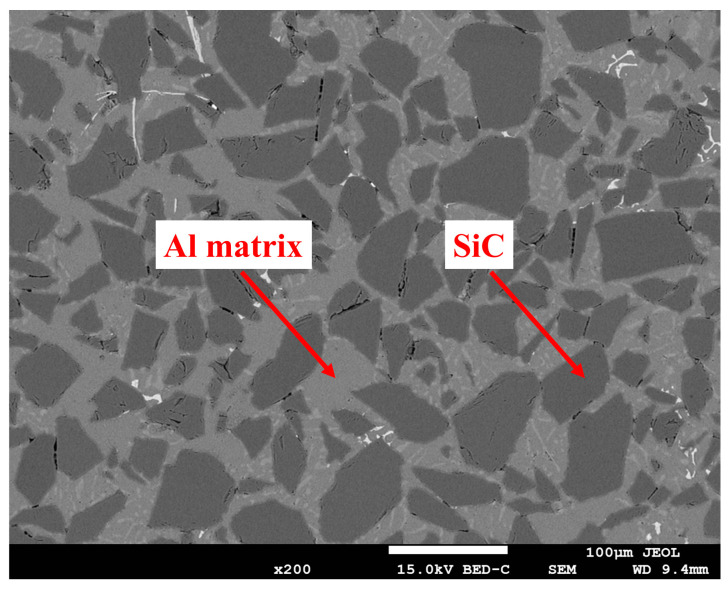
Micromorphology of55% SiCp/Al composite.

**Figure 2:**
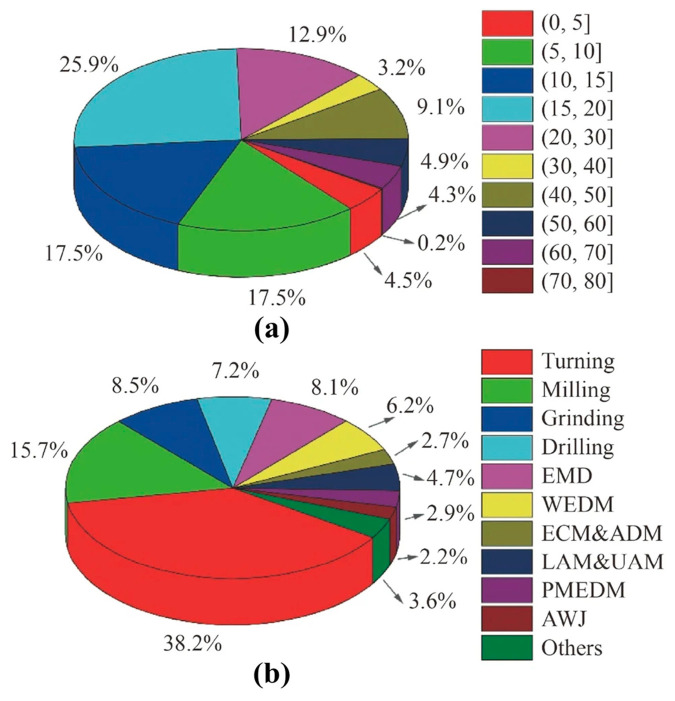
(**a**) Percentage of studied SiC fractions and (**b**) distribution of machining methods [5].

The SiCp/Al composite materials exhibits different material properties due to the difference in SiC particle volume fraction. The specific performance parameters are shown in Table 1. The microstructure (55% volume fraction) of the materials obtained from the experiment is shown in Figure 1 [1,2,3,4]. Due to the hard brittleness of SiC particles, machining this material is challenging. Therefore, numerous scholars have researched it to improve its machinability. Based on the database organization, the distribution of different SiC volume fractions and processing methods is shown in Figure 2a and Figure 2b, respectively. It was found that the relevant research mainly focuses on low-volume-fraction SiCp/Al composite materials and conventional processing methods (turning, milling, grinding, drilling). Moreover, the performance parameters of SiCp/Al composite materials vary greatly with different volume fractions, further complicating the processing. To address this, this review examines both conventional and unconventional processing studies to assess the current status and shortcomings of SiCp/Al processing, achieving efficient and high-quality processing of SiCp/Al.

## 2. Machining Mechanism of SiCp/Al

The precision machining of high-hardness particle-reinforced metal matrix composites is facing significant challenges. Not only does it exhibit poor machining performance but it also entails a lengthy process for subsequent surface treatment. Therefore, scholars have conducted extensive research, and numerical research has garnered increasing attention due to its advantages, including low cost, high efficiency, low time consumption, and the ability to delve into intricate machining details, like tool particle interaction and chip removal mechanisms.

**Figure 3:**
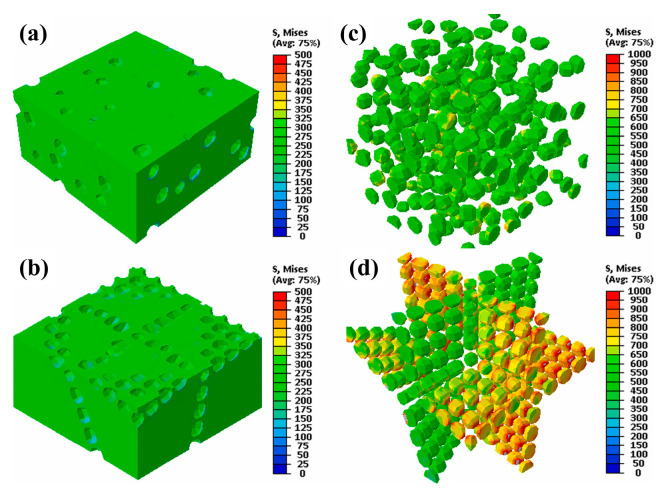
Load-bearing state of reinforcement and matrix in SiC/Al composite with homogeneous distribution (**a**,**b**) and network architecture (**c**,**d**) near a yield strain εxx = 0.4%. (**a**,**b**) Load-bearing states of Al alloy matrix; (**c**,**d**) Load-bearing states of SiC particles. [Reproduced with permission from Gao et al.; published by Elsevier, 2019] [16].

The accuracy of finite element modeling is essential for studying the machining mechanism of SiCp/Al composite materials. Chen et al. [6] compared the cutting mechanism of SiCp/Al using uniformly distributed particles of the same size at the micro-level and randomly distributed non-equally sized particles. A uniform model of the same size can obtain good stress distribution and chip morphology. However, random models can more accurately simulate chip cutting, workpiece cutting force fluctuations, and workpiece surface damage. Chen et al. [7] revealed the mechanism of chip formation in SiCp/Al6063 composite materials through a three-dimensional finite element end-milling model and an equivalent homogeneous material (EHM) model. The improved Johnson–Cook constitutive model based on strain gradient theory by Fan et al. [8] effectively reflected the size effect of workpiece materials. Wang et al. [9] studied the fracture and removal mechanism of SiC particles and their impact on surface formation, finding that the particle fragments squeezed by the cutting tool during the cutting process changed the mechanism of chip formation. Du et al. [10] analyzed the removal modes of reinforced particles and Al matrix and their effects on surface formation mechanisms through the simulation of single diamond abrasive cutting. It is believed that when processing SiCp/Al composite materials, SiC is removed through methods, such as crushing, debonding, fracture, shearing, pulling in, and pulling out. Zhou et al. [11] proposed a deformation mechanism and surface quality finite element model that accurately predicted metal matrix composites (MMCs). They found that serrated chips mainly form along the shear plane, accompanied by the generation and propagation of microcracks. Wang et al. [12] discovered complex chip formation mechanisms through high-speed milling simulations, including matrix deformation and fracture, debonding between the matrix and SiC particles, and the potential fracture of some particles. Zhang et al. [13] studied the existence of microcracks in the material preparation process based on composite fracture mechanics. Song et al. [14,15] obtained the following through simulation research: the fracture toughness and tensile ductility of composites decrease with the increase in the SiC particle volume fraction. As shown in Figure 3, Gao et al. [16] compared composite materials with a network system structure and a homogeneous structure and found that SiC particle walls parallel to the external load direction have higher load-bearing capacity, while walls perpendicular to the load direction exhibit similar stresses to homogeneous composite materials.

Cutting technology is primarily a mechanical processing method that utilizes cutting tools to remove excess material from the surface of a material in order to achieve the desired shape, size, and surface finish of the workpiece. The cutting process is highly complex, thus necessitating in-depth research on the cutting mechanism and chip formation theory. This research is essential for establishing and developing effective cutting models that can quantitatively predict relevant process parameters in the cutting process, and optimize the cutting conditions to improve processing efficiency is of great significance. Shui et al. [17] simulated the cutting of two-dimensional randomly distributed circular particle models, circular mixed regular polygonal particle models, and arbitrary polygonal models with a volume fraction of 30%. It was observed that the cutting force impact frequency was lower for composite materials with uniformly distributed small circular particles compared to the other two models, resulting in a relatively stable cutting process. Furthermore, the interaction between polygonal particles and cutting tools is stronger than that of circular particles. The interaction between particles, matrix, and cutting tools could generate pits on the machined surface. Wang et al. [18] established a high-volume-fraction randomly distributed circular SiC particle model and a polygonal SiC particle model. Similar conclusions have been drawn in terms of cutting force and machined surface quality. Yin et al. [19] first established a friction coefficient model that considers the effects of temperature and particle characteristics. The impact trends of temperature, particle volume fraction, average particle size, and pressing depth on the friction coefficient of various SiCp/Al composites were evaluated. Jiao et al. [20] simulated the high-speed milling process of a SiCp/Al metal matrix composite based on deformation and studied the influence of the given cutting parameters on cutting temperature and tool wear. Cutting speed has the most important impact on cutting temperature and tool wear.

Further research on the cutting mechanism of SiCp/Al involves equating the model with the tool and single particle cutting. Liu et al. [21,22] conducted similar research on 45% SiC/Al composite materials and discovered that the micro-milling feed rate per tooth is approximately half the size of the particle, resulting in a relatively good cutting surface. Lu et al. [23,24] suggested that the interaction between the tool and particles in the lower and middle parts of the particles can yield a smooth machined surface. Additionally, the rake angle of the cutting tool significantly changes the established stress state in the tool particle contact area, leading to a strong coupling effect between the rake angle of the tool and the position of the tool particle in SiCp/Al diamond cutting. Rashid et al. [25] employed an ellipse as the geometric shape of SiC particles and simulated three different positions of SiC particles relative to the cutting tool path.

The scratch test is not only helpful in analyzing the micro-mechanical properties of materials but also holds significance in studying material processing mechanisms and processability. To gain a deeper understanding of the properties of SiCp/Al composite materials, scholars conducted scratch tests on them. Among these studies, Zhao et al. [26] compared the removal mechanisms of SiCp/Al, SiC ceramics, and Al alloy and discovered that the material removal behavior of SiCp/Al at the macroscopic scale is similar to that of Al alloy, albeit with minor differences. However, at the microscopic scale, it is fundamentally different from the other two materials, primarily due to the coupling effect between SiC particles and the Al matrix. Yan et al. [27] observed that the scratching process consists of friction, plowing, plastic cutting, and SiC fracture. Considering the size effect, the influence of SiC particle contrast energy is related to the ratio of the material volume fraction to particle radius. Although larger cutting depth can maintain a smaller specific energy, it has a greater impact on material removal. This is because a larger scratching depth directly increases the probability of particle–particle interaction, leading to more cracks and pits on the material surface [28].

**Figure 4:**
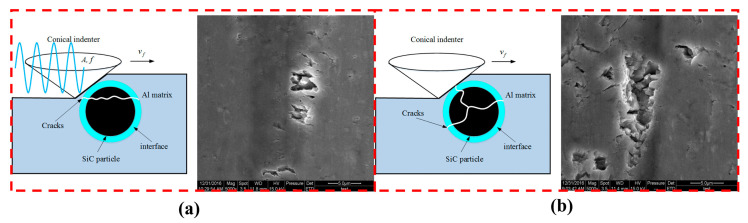
The material removal mechanism of SiC particles in US (**a**) and TS (**b**) tests. [Reproduced with permission from Zheng et al.; published by Elsevier, 2018] [31].

With the application of ultrasonic vibration-assisted processing technology, scholars have shown great interest in studying the material removal mechanism of this processing technology. Ultrasonic vibration-assisted scratching can achieve plastic removal of materials at large cutting depths, and increasing the ultrasonic amplitude can increase the material removal rate [29]. Furthermore, the scratching load and friction coefficient are relatively small, while the depth and width of the scratches are relatively large [30]. In their study on SiCp/Al composite materials, Zheng et al. [31] found that the scratching profile surface was relatively smooth, and the edges were relatively intact. Surface defects mainly appeared as grooves caused by the fracture and breakage of SiC particles, rather than large pits caused by the complete removal of the entire particles. Additionally, due to the obstruction of SiC particles to the plastic flow of the aluminum matrix, microcracks may appear on the surface, and there are also many small SiC fragments in the chips. Therefore, the degree of SiC particle fragmentation determines the surface integrity, and a lower scratching speed will exacerbate the degree of SiC particle fragmentation, thus indirectly enhancing surface integrity [32]. Figure 4 illustrates the material removal modes of SiC particles in ultrasonic scratching (US) and traditional scratching (TS) tests.

**Figure 5:**
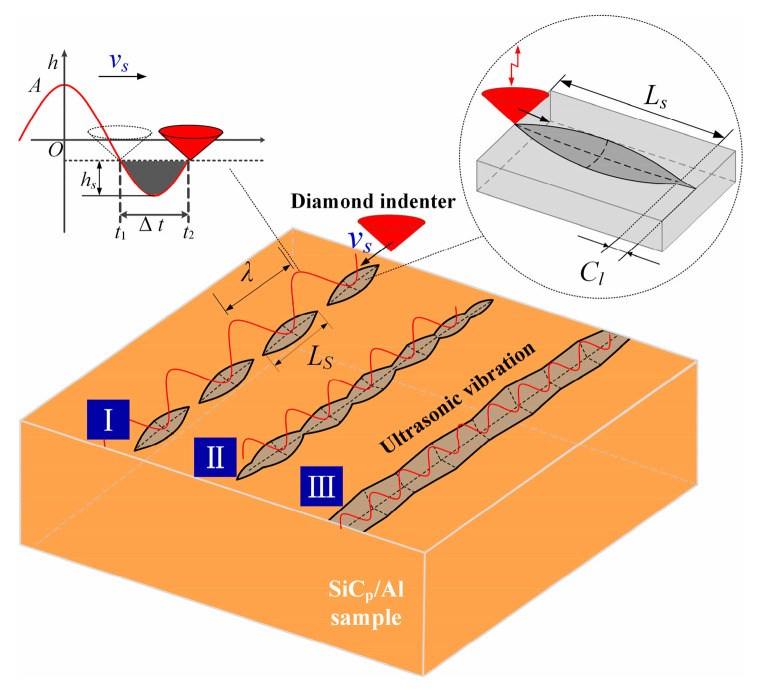
Three types of scratch trajectories. [Reproduced with permission from Li et al.; published by Elsevier, 2023] [33].

**Figure 6:**
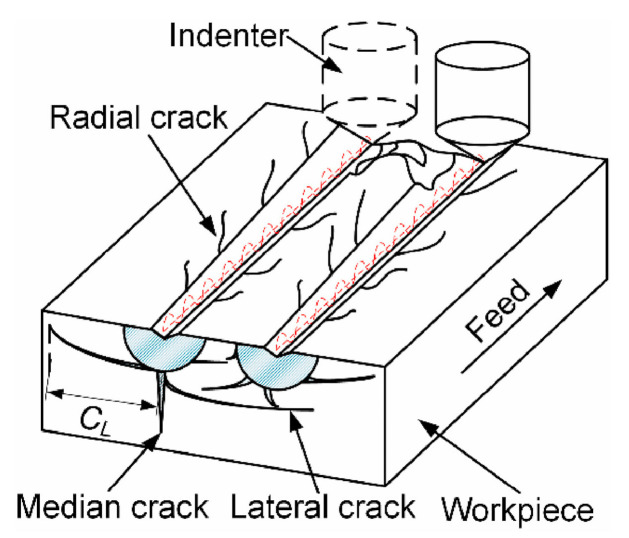
Double-scratch model. [Reproduced with permission from Qiao et al.; published by Elsevier, 2022] [34].

Li and his team [33] conducted research on three types of scratch trajectories: continuous, semi-continuous, and intermittent (as shown in Figure 5). The study found that the trajectory pattern is influenced by the scratch speed, scratch depth, ultrasound amplitude, and frequency. The intermittent scratch pattern should be avoided in actual processing, while the continuous scratch pattern can achieve better surface integrity. Qiao et al. [34] conducted a dual-scratch experiment with different scratch depths and spacing distances, as shown in Figure 6. Increasing the spacing distance leads to crack propagation, and ultrasound vibration helps promote the expansion of shallow transverse cracks and reduce the depth of crack damage.

To fully maximize the benefits of ultrasonic vibration-assisted machining, it is crucial to ensure that the processing parameters align with the vibration frequency and amplitude. To optimize the reciprocating polishing behavior and enhance intermittent cutting characteristics, a high overlap rate needs to be achieved. This is carried out with the aim of reducing low cutting forces and enhancing surface quality [35].

In summary, SiC particles are primarily eliminated through processes, such as crushing, debonding, fracture, and pressing. The workpiece’s surface exhibits defects, such as large and small pits, protrusions, and scratches. Additionally, there are substantial fluctuations in stress, cutting force, and cutting temperature during the processing. The chips formed are mainly serrated, and the quality of the workpiece processing is greatly influenced by the process and material parameters. Enhancing the understanding of the processing mechanism of SiCp/Al composite materials can assist in enhancing and surpassing the processing performance of SiCp/Al composite materials.

## 3. Turning Machining of SiCp/Al

SiCp/Al composite materials present challenges in machining, as it is difficult to ensure both processing efficiency and quality simultaneously. Precision and ultra-precision turning techniques can address this issue by enhancing processing efficiency, reducing costs, and ensuring the surface and end-face processing quality of rotating parts.

The poor surface quality of MMC processed by SiCp/Al composite material is due to the low plasticity and non-uniformity of the reinforced particles. This results in severe tool wear [36], including rake wear, flank wear, and edge wear; flank wear is the primary wear mode, with adhesive wear and abrasive wear as the primary wear mechanisms [37]. Polycrystalline diamond (PCD) has proven to be effective in processing abrasive systems such as aluminum/silicon and glass-filled polymers. It offers advantages in composite material processing but also experiences wear similar to other materials, such as adhesive wear and fatigue fracture [38]. Diamond coatings appear to be more economically feasible than PCD for MMC machining, with the main wear mechanism being coating failure caused by high stress [39]. In composite material processing, the main mechanism leading to tool wear in TiN-coated tools is abrasive wear [40], while multi-coated hard alloy tools outperform double-coated tools in terms of wear performance [41]. When processing small-size SiC particle composites, cubic Boron nitride (CBN) tools suffer mainly from flank wear, while for large-size SiC particle composites, the cutting edge and tooltip experience severe breakage, making CBN tools unsuitable for MMC processing [42]. In summary, PCD tools possess a long lifespan and excellent machining performance, making them the preferred choice for MMC machining [43].

Process parameters significantly affect tool wear. Under dry turning conditions, cutting speed primarily influences tool wear and increases with higher cutting speeds [44]. Increasing the volume fraction and size of SiC particles also leads to increased tool wear [45]. Larger SiC particles impose a higher impact strength on the tool, resulting in micro-breakage extending to the back face and complete damage to the cutting edge [46]. The increase in feed speed and cutting depth can contribute to an increase in tool flank wear, but the impact is relatively minor [47].

Tool wear predominantly occurs on the flank surface, necessitating the prediction of tool wear. Researchers such as Saeed [48] and Rashid et al. [49,50] have employed turning experimental devices and artificial neural networks (ANNs) to predict the lateral wear of the tool. They analyzed the influence of process parameters on tool surface wear. Additionally, Rajesh et al. [51] obtained optimal solutions for tool wear and the material removal rate (MRR) by combining experimental data with mathematical analysis methods. After tool wear, the machining accuracy of the workpiece diminishes, surface roughness increases, cutting force and temperature rise, and even vibration may occur. Therefore, tool wear can indirectly manifest through cutting force, temperature, and surface roughness.

**Figure 7:**
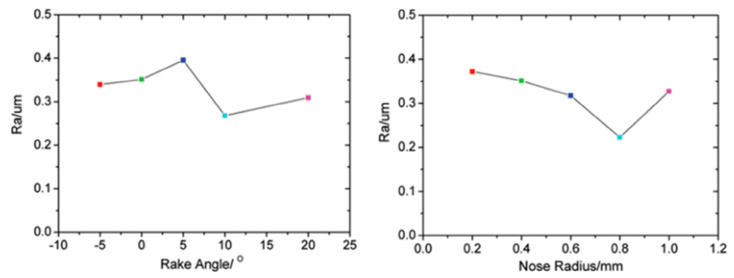
Variation of surface roughness with tool rake angle and tool nose radius. [Reproduced with permission from Dong et al.; published by Taylor & Francis, 2013] [53].

The fracture and removal method of SiC particles within the material directly impacts surface quality, with surface roughness aligning with the standard radius of SiC particles [52]. Hence, larger SiC particles yield poorer machined surface quality. Tool wear directly affects the quality of the machined surface. Dong et al. [53] investigated the influence of tool rake angle and tooltip radius on surface roughness through turning experiments, basing their study on the geometric shape of the tool. In Figure 7, a large rake angle can improve surface quality, but an excessive rake angle can accelerate tool wear. However, an increase in the edge radius significantly enhances surface quality. Tool wear can also lead to surface damage depth in subsequent processing, affecting the fatigue performance of composite material structures [54,55].

With continuous improvements in performance requirements for SiCp/Al material components and devices, there is an urgent need for high-quality surfaces. Ultra-precision machining technology is usually required to produce surfaces with nanoscale roughness and extremely high quality. Single crystal diamond tool ultra-precision turning (SPDT) technology is a machining technique that utilizes single crystal diamond tools with nanoscale edge radii and excellent wear resistance for turning. Many materials can be directly turned into optical surfaces through SPDT technology without the need for subsequent grinding and polishing. Ge et al. [56,57,58] observed that extraction, cutting, crushing, and pressing are the main four removal mechanisms of SiC particle-reinforced composite materials. During turning, serrated chips are generated, resulting in a surface roughness of Ra = 20–30 nm. The deformation layer caused by processing extends to 8–16 µm below the machined surface. Additionally, the processing performance of SCD is better than that of PCD tools [59]. Lu et al. [60] found that the number of cutting steps has a significant impact on the interaction between particles and cutting tools, as well as on the quality of the processed surface.

Therefore, when machining MMC materials in rough and medium turning, it is recommended to choose tools with low cutting speed, high feed rate, and cutting depth. Secondly, to ensure a longer tool life, it is necessary to choose tools with smaller tool rake angles. When precision turning, it is recommended to choose tools with high cutting speed, low feed rate, cutting depth, and larger tool rake angles and tip radii to ensure better surface quality.

## 4. Milling Machining of SiCp/Al

As the processing morphology of SiCp/Al becomes increasingly diverse, milling has rapidly developed due to its advantages [61,62]. In theory, milling technology can achieve surface roughness and geometric accuracy in the range of tens of nanometers to micrometers [63,64]. Therefore, milling technology can be considered for the processing of composites to achieve the flexible and precise machining of composite parts.

The processing adaptability and tool wear characteristics of different tool materials vary greatly. By comparing the wear characteristics of cermet tools, TiN-coated tools, and cemented carbide tools when milling large-particle high-volume fraction SiCp/Al composites, it is found that the tool wear of these three tools is mainly flank wear, and the wear resistance is almost the same. The tool wear rate increases with the increase in cutting speed [65]. The wear characteristics of different tools under dry and wet processing conditions also vary greatly. Although tool wear is mainly manifested as side wear, the tool wear rate is relatively low under wet processing conditions, and the surface quality is relatively good. The performance is more pronounced in the case of long-cutting distances [66]. Yang et al. [67] believed that the appropriate grain size of PCD tools can improve the surface quality of machining and reduce tool wear, which mainly depends on the size, shape, and distribution of SiC particles. However, PCD tools with a certain grain size are difficult to achieve both good surface quality and minimal tool wear. PCD tool life is related to grain size and composite material parameters. Huang et al. [68,69,70] investigated the use of PCD tools with different grain sizes (5, 10, 25, 32 μm). In the relevant research on milling SiCp/Al composites with different volume fractions (15, 25, 30, 56%), it was concluded that when cutting SiCp/Al composites with larger SiC particles, PCD tools with smaller diamond grains have better wear resistance. On the contrary, when cutting smaller SiC particle composite materials, PCD tools with larger diamond grains have better wear resistance. Wang et al. [71] found that the higher the milling speed, the shorter the tool life. The coarser the PCD grain, the higher the wear resistance, so the optimal particle size of PCD was determined to be 10 µm.

Flank wear and fragmentation are the two main types of tool wear. The wear mechanisms of PCD tools are abrasive wear and adhesive wear [72]. Deng et al. [73] proposed an analysis method that combines two-body/three-body abrasive wear and adhesive wear. In the initial processing stage, adhesive wear dominates, while in the later processing stage, two-body abrasive wear becomes the main tool flank wear mechanism. A smaller feed rate per tooth leads to more severe tool flank wear. High spindle speed and a small tool clearance angle can also lead to more tool side wear. Han et al. [74] studied tool wear during milling based on the geometric structure of the main cutting edge. It was found that the processing technology of PCD tools with chamfers is different from that of PCD tools with sharp edges. The chamfered edge can effectively reduce the friction between the rake face and chips, thereby extending the service life of the tool.

The influence of cutting process parameters on cutting force and surface quality has also been studied by many scholars. Among them, the degree of influence on milling force, in descending order, is cutting depth, feed rate, and cutting speed [75]. The cutting force and cutting temperature increase with the increase in feed speed or radial cutting depth. In terms of cutting force, it is recommended to use a negative rake angle and a relatively large tooltip radius. Tools with a positive rake angle or a larger edge radius produce lower cutting temperatures [76,77,78]. Secondly, cutting parameters have a significant impact on the depth of large pits on the machined surface. High feed speed and low cutting speed correspond to large cutting forces and result in deep pits [79]. The increase in feed per tooth deepens the thickness of the fractured SiC Stratum granulosum under the surface, which aggravates the surface defects [80]. By setting appropriate milling parameters, a mirror surface with lower surface roughness can be obtained [81]. The tooltip radius determines the machining performance. As the radius of the tooltip increases, the chips become shorter and wider. It was also observed that the cutting force increases with the increase in the tooltip radius. As the radius of the tooltip increases, outer-edge defects will become slight peeling and ductile fractures, and the quality of the inner and outer edges of the material will be improved [82]. In addition, when high-speed milling is performed on thin-walled specimens of high-volume-fraction SiCp/Al composite materials, the effect of increasing milling speed on the surface roughness of the cutting inlet, middle part, and cutting outlet shows a decreasing trend. The Ra value at the cutting inlet is the smallest, followed by the middle, and the Ra value at the cutting outlet is the highest [83]. Therefore, by selecting higher cutting speeds, smaller radial cutting depths, and a moderate feed per tooth, the cutting force can be reduced. It is also important to select the appropriate milling mode to reduce edge defects, thereby improving the machining efficiency and accuracy of the workpiece [84,85].

By optimizing cutting parameters, a higher efficiency can be achieved in milling composite materials with different volume fractions, resulting in improved surface quality and tool life. Neural networks and Taguchi methods [86], analysis of variance [87], and genetic algorithms [88] are some of the parameter optimization methods used. Table 2 provides a compilation of recommended parameters for milling SiCp/Al composite materials.

It is important to establish appropriate standards to evaluate the quality of processed surfaces. Wang et al. [98] suggested that three-dimensional parameters such as Sa and Sq are better able to describe the impact of milling parameters on surface quality, with a preference for Sq due to its sensitivity. Sq decreases with milling speed and increases with feed speed. Both 3D roughness Sq and PSD are effective in characterizing the machined surface microstructure of SiCp/Al composite materials [99,100,101]. Liu et al. [102] evaluated the surface topography through 3D surface roughness (Sq) and fractal dimension (Ds). Sq is mainly affected by milling width and depth, while Ds is mainly affected by milling width. There is a weak negative correlation between them. Ds is more sensitive to major defects, and, generally, larger Ds corresponds to good surfaces.

In summary, for high-speed milling of SiCp/Al composites, it is recommended to use PCD tools with larger rake angles and tip radii. Additionally, larger milling speeds and smaller milling depths are advised to achieve efficient and high-quality surface milling.

## 5. Drilling Machining of SiCp/Al

SiCp/Al composite materials face challenges during drilling due to the presence of hard particles in the matrix. While efforts have been made to develop near-net shape manufacturing for these products, precision machining is still required for the assembly process. Drilling is typically the final manufacturing process conducted before assembly, making it crucial to understand the drilling process in MMCs for selecting appropriate tool materials and producing high-quality holes.

The drill bit exhibits lateral wear, with the main wear mechanisms being abrasive wear and lateral adhesive wear [103]. Secondly, predicting drilling force is of great significance in improving drilling efficiency and optimizing drilling parameters. Liu et al. [104] proposed, for the first time, an energy-based mechanical modeling method to predict cutting forces during the drilling process of SiCp/Al composite materials. The energy consumed by the action of abrasive particles is estimated to be 8.2–13.6% of the total cutting energy. The energy consumed by the action of abrasive particles increases with the increase in the particle volume fraction and feed rate. Zhu et al. [105] developed a drilling force testing system and established a prediction model for high-volume-fraction SiCp/Al drilling force based on an improved BP neural network algorithm. The influence of drilling process parameters on drilling force was determined.

To enhance drilling efficiency, reduce costs, and improve the quality of drilled surfaces, it is necessary to optimize drilling conditions [106]. Davim et al. [107] conducted a drilling experiment on granular MMC. It was observed that the surface finish of drilled sample holes deteriorates as the feed rate increases, while there is no significant change when altering the cutting speed. S. Basavarajappa et al. [108] identified that the feed rate is the primary factor influencing the thrust of aluminum-based silicon carbide composites, while the cutting speed and its interaction with feed rate have the smallest impact. Kesen et al. [109] utilized gray correlation analysis to evaluate and optimize the drilling processing parameters of particle-reinforced metal matrix composites (MMCs). Through a drilling experiment, it was discovered that the content fraction is the most influential factor on the gray level, followed by the drill material, feed rate, and spindle speed. However, there is a notable disparity in drilling situations between thin-walled and thick-walled workpieces. The drilling of thin-walled workpieces is sensitive to changes in feed rate in terms of drilling force and torque. This is due to the significant impact of feed rate on the concave deformation and critical thickness of thin-walled workpieces, resulting in cracking [110].

The factor affecting the quality of drilling is the tool material. Ramulu et al. [111] conducted drilling studies on composite materials using different drill bits (high-speed steel, hard-alloy drill bits, and PCD (drill bits)). It was found that PCD drill bits not only have the highest wear resistance but also outperform the other two drill bits in terms of drilling quality and minimum drilling force. Chen et al. [112] found that the defect at the entrance edge is the result of SiC particle peeling and fracture, and exit-edge defects are formed by extended peeling. Due to the large force applied to the uncut workpiece, the defect at the exit edge is more severe than at the entrance edge. The wear mechanism of PCD drill bits mainly includes abrasive wear and micro tilting. Xiang et al. [113] developed a diamond tool wear model for studying the evolution of tool wear.

The research organization of some scholars is shown in Table 3.

In summary, when drilling thick-walled or thin-walled SiCp/Al composite workpieces, priority should be given to using PCD tools and ensuring that the feed speed is as low as possible. This not only reduces the frequent replacement of worn tools to improve drilling efficiency but also ensures the surface quality of the drilling.

## 6. Grinding Machining of SiCp/Al

The poor grindability of aluminum alloys and the hard brittleness of SiC particles pose a challenge in grinding SiCp/Al composites [119,120]. Numerous scholars have conducted extensive research on the grinding mechanism and the impact of grinding parameters on the grinding force and surface roughness [121].

Sahin et al. [122] found that SiCp/Al exhibits better wear resistance compared to many difficult-to-machine materials. This finding further highlights the challenges associated with processing SiCp/Al. The grinding process of SiCp/Al involves four stages of material removal: plastic removal of the aluminum matrix, crack initiation of SiC particles, crack propagation of SiC particles, and brittle fracture of SiC particles [123]. The removal of SiC particles primarily occurs due to interface failure between the reinforcement and matrix, resulting from microcracks on the interface and numerous fractured or crushed SiC particles on the surface. Brittle fracture is the main removal mechanism for SiC particles; only a few particles are pulled out [124]. The chips formed during this process can take on four shapes: curved, curled, flake, and ribbon; flake chips are the most common. The mechanism of chip formation heavily relies on the plastic deformation of the aluminum matrix, the contact position between SiC particles and diamond particles, and the method of SiC particle removal [125,126]. Moreover, a smaller SiC particle size leads to higher surface quality and accuracy in the final SiCp/Al product [127]. Additionally, a higher proportion of particles removed through shear results in fewer particles being removed through fracture, ultimately improving the machined surfaces [128].

As the size of sand particles decreases, the amount of space available for storing debris decreases, making it easier to overload and making the precision machining of surfaces more difficult [129]. Employing electrolytic online wheel dressing (ELID) grinding technology [130,131,132,133] can reduce grinding defects and achieve a high-quality surface. ELID grinding can effectively reduce the proportion of SiC particles that undergo brittle fracture, ensuring that SiC particles are removed in a ductile manner, resulting in a surface of high integrity for SiCp/Al composite materials [134]. In a comparison conducted by Shanawaz et al. [135], it was observed that the grinding force generated by ELID grinding is higher compared to traditional grinding. The researchers concluded that the current duty cycle is a significant influencing factor in ELID grinding. When the current duty cycle increases, the cutting force proportionally decreases, and the surface roughness correspondingly increases. Guan et al. [136] achieved a lower machined surface roughness of Ra30nm through precision grinding experiments. The recommended process parameters are a spindle speed of 1500 r/min, a grinding depth of 0.1 μm, a sample movement speed of 4 m/min, and a voltage of 15 V.

Different grinding conditions can result in various grinding effects. Huang et al. [137] conducted grinding experiments on SiCp/Al composites using wet, dry, low-temperature, and ELID grinding conditions. Among the four conditions, the grinding force is the highest under low-temperature conditions, followed by the cutting force under dry conditions, and the milling force under wet conditions is the lowest. Moreover, for wet and dry conditions, the grinding force ratio decreases as the grinding depth increases, while for low-temperature and ELID grinding conditions, the grinding force ratio slightly increases with an increase in grinding depth. Zhou et al. [138] performed experimental research on the grinding characteristics of SiCp/Al composite materials under low-temperature and wet conditions using surface grinding technology. The use of low-temperature cooling effectively enhances the support effect of the aluminum matrix on SiC particles and improves surface quality. Additionally, it suppresses the brittle fracture of SiC particles, and the observation of ductile stripes on the SiC particles is possible.

The optimization of grinding parameters can not only improve surface quality but also enhance machining efficiency. The spindle speed has the greatest impact on surface roughness, followed by grinding depth and feed speed [139]. Furthermore, the grinding force generated during the grinding process decreases with an increase in spindle speed, while it increases with higher feed speed and grinding depth [140]. The influence of grinding depth on the grinding force surpasses that of feed speed. Therefore, to ensure a certain material removal rate, it is advisable to increase the cutting speed and feed rate to improve the surface quality of grinding [141]. Resin-based diamond grinding wheels exhibit superior surface quality compared to ceramic-bonded and electroplated diamond grinding wheels [142]. Zhao et al. [143] conducted grinding experiments on SiCp/Al composite material samples with a high-volume fraction and extensively examined the influence of machining parameters on surface quality. They recommend reducing the feed rate, increasing the cutting depth, and elevating the spindle speed during the grinding process to strike a balance between grinding efficiency and surface quality [144].

**Figure 8:**
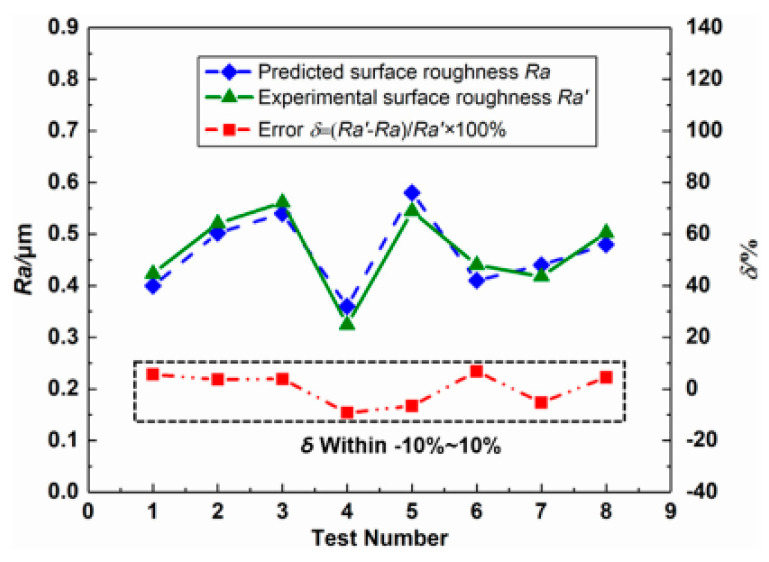
Predicted and experimental surface roughness. [Reproduced with permission from Zhu et al.; published by Elsevier, 2019] [153].

Establishing a corresponding theoretical model holds importance in guiding grinding processing. It has been observed that the grinding temperature gradually decreases with an increase in workpiece speed, while the grinding temperature increases with an increase in grinding depth. Zhang [145], Zhou [146], and others confirmed this pattern through a triangular heat source model, with the highest grinding temperature also following the same pattern [147]. Furthermore, Lu et al. [148] conducted experiments and demonstrated that the predicted deviations for axial force and torque are 7.8% and 5.2%, respectively. Additionally, Yin et al. [149] proposed an analytical grinding force model for grinding SiCp/Al composite materials. Their findings revealed that grinding parameters have similar effects on surface roughness and grinding force. This model can be utilized to optimize process parameters and enhance grinding quality. Kwak et al. [150] employed a response surface model and genetic algorithm to optimize grinding parameters for aluminum-based metal matrix composites. The optimization of grinding parameters through GA and RSM proves to be highly effective and feasible in achieving better surface roughness and minimizing grinding force. Wang et al. [151,152] established stability analysis models for the machining process of SiCp/Al composite thin-walled workpieces, along with dynamic models and criteria for flutter analysis. Simulation results verified that the model can optimize the selection of processing parameters for RUM, effectively preventing chatter and achieving desirable processing outcomes. Zhu et al. [153] proved that the exponential composite function is the most suitable. Additionally, a comprehensive evaluation system was established, which intuitively reflects surface morphology and describes surface voids and defects, as depicted in Figure 8. Experimental verification confirmed the feasibility of this approach. The optimal grinding parameters determined are as follows: grinding wheel speed of 25 m/s, workbench speed of 1.437 m/min, and grinding depth of 3.857 μm. The removal rate is 2.771 mm^3^/s [154].

When grinding high-volume-fraction SiCp/Al to achieve a better surface finish and accuracy, the first step is to select a grinding wheel with a fine abrasive size. Additionally, it is essential to ensure that the processing is set with an appropriately high spindle speed, lower feed rate, and grinding depth.

## 7. Special Machining of SiCp/Al

The use of conventional processes, such as milling, turning, drilling, and grinding, to process SiCp/Al composite materials often results in excessive tool wear and poor surface finish. This is due to the significantly higher hardness of SiC particles compared to the metal matrix. Consequently, unconventional techniques have also been implemented for these materials [155,156].

EDM/WEDM is a non-contact material removal process that is not limited by the strength and hardness of the material, making it suitable for meeting processing requirements. This process utilizes a significant amount of concentrated heat energy generated by discharge sparks to melt and evaporate the materials of the workpiece [157,158,159,160,161]. Moreover, EDM/WEDM has been proven to be an effective method for processing conductive materials with varying degrees of hardness [162]. Material removal rate (MRR) and surface roughness (Ra) serve as two essential performance indicators to assess machining efficiency and quality [163]. Additionally, the tool wear rate (TWR) is a crucial indicator for EDM/WEDM. For electrical discharge machining (EDM) of MMC, Kumar et al. [164] proposed a low-temperature cooling electrical discharge machining method (CCEDM) for machining 10 wt% SiCp/al.The proposed low-temperature electrical discharge machining not only enhances the material removal rate by 39.8% but also reduces electrode wear by 18% and surface roughness by 17–29%. Hu et al. [165] conducted a comparative study on the surface performance of powder-mixed electrical discharge machining and conventional electrical discharge machining. They concluded that the surface quality of powder-mixed electrical discharge machining improved substantially, reducing the roughness by approximately 31.5%.

Micro electric discharge wire cutting, as proposed by Chen et al. [166], not only has the capability to stably process high-volume-fraction composite materials but also addresses the issue of abnormal wire breakage that occurs during the processing. This technique achieves a maximum material removal rate (MRR) of 0.261 mm^2^/s, while producing a minimum surface roughness of 3.6 μm. In order to ensure both efficient machining and a satisfactory machining surface, it is recommended to adjust the short pulse on time, long pulse off time, line speed, and servo voltage accordingly. Additionally, it is advised to develop a mathematical model for surface roughness (SR) in order to determine the optimal combination of machining parameters [167]. Dev et al. [168] further investigated the feasibility of micro electrical discharge machining for composites with high-volume fractions, using rotating tube electrodes. Despite the low conductivity and high thermal resistance of SiC-reinforced particles, the results obtained indicate that effective processing of SiCp/Al composites can be achieved.

EDM machining of PRMMC faces serious issues, including severe tool wear and difficulty in debris removal. Furthermore, EDM can cause significant surface damage to materials, leading to the destruction of microstructure and resulting in poor surface integrity [169]. These factors greatly restrict the application of PRMMC in EDM processing. Thus, it is crucial to investigate the corrosion mechanism of SiCp/Al composites in electrical discharge machining and explore the impact of machining parameters in order to enhance the machining performance further.

**Figure 9:**
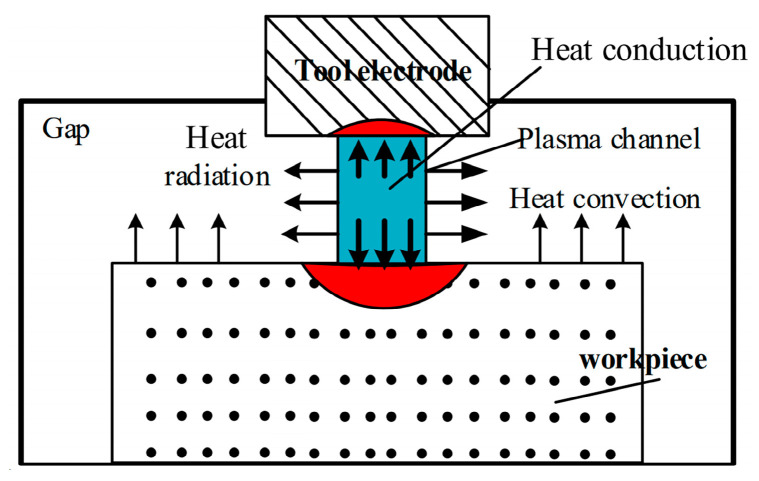
Schematic diagram of the SiCp/Al composite material EDM [171].

Seo et al. [170] studied the machinability and processing quality of high-volume-fraction composite materials using EDM. They observed that the material removal rate increased with increasing peak current and pulse on time until reaching the optimal point, and then they sharply decreased. Liu et al. [171] discovered that increasing the pulse width initially decreased and then increased the temperature in the workpiece during the ablation process of single-pulse electrical discharge machining. Furthermore, they observed that the temperature increased with the increase in peak current. Appropriately reducing the current would be beneficial for efficient discharge during the treatment process. Figure 9 illustrates a schematic diagram of EDM.

**Figure 10:**
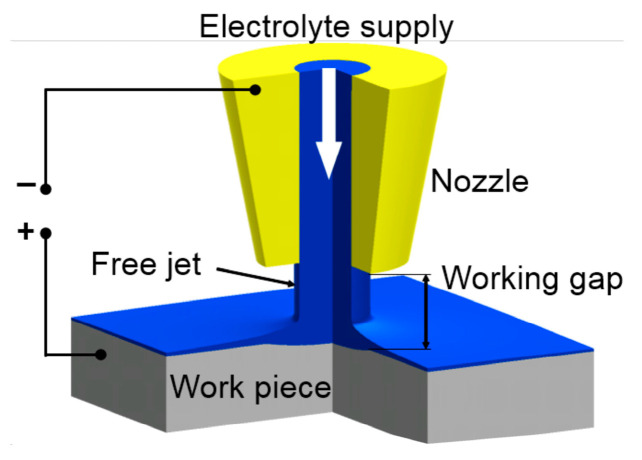
Principle of Jet-ECM [173].

In addition to electrical discharge machining, numerous scholars have also conducted studies on electrochemical jet machining. Norbert et al. [172] utilized three pH-neutral water electrolytes—sodium bromide, sodium chloride, and sodium nitrate—to perform electrochemical jet machining on SiCp/Al. The principle can be seen in Figure 10. The dissolution characteristics vary depending on the electrolyte utilized. The deepest point erosion is achieved by using NaCl as the electrolyte, followed by NaBr, while the shallowest erosion is obtained with NaNO_3_ or sodium bromide. Moreover, the width of point erosion primarily relies on the diameter of the nozzle employed. Therefore, the achievable width ranges from 2- to 3-times the nozzle diameter. The applied voltage and processing time are the main factors that control the size of point corrosion. The influence of particle fraction on depth and width is relatively minor.

**Figure 11:**
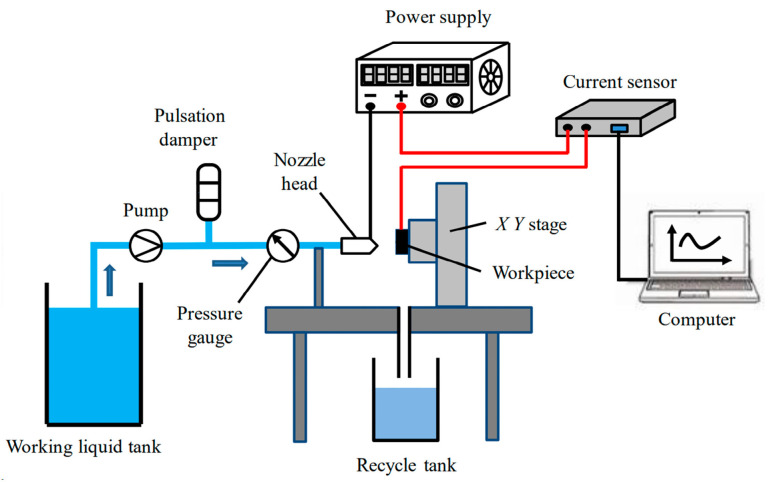
Schematic of experimental apparatus [175].

Liu et al. [174] also conducted blind hole drilling experiments on 65% SiCp/Al composite materials using a NaCl electrolyte as the working fluid. In Figure 11, it was observed that the inlet diameter of the processed hole is approximately 3–4-times the spray size. Moreover, a comparison was made on the removal mechanism and surface quality of abrasive-assisted electrochemical jet machining (ECJM) [175]. The study revealed that the material removal mechanism involves substrate corrosion and extraction of SiC particles. The pits formed due to the extraction of SiC particles and the SiC protrusions caused by substrate corrosion significantly impact the roughness of the machined surface. The surface roughness resulting from ECJM and abrasive-assisted electrochemical jet micromachining (AECJM) ranges from Ra 10 to 16 μm.

**Figure 12:**
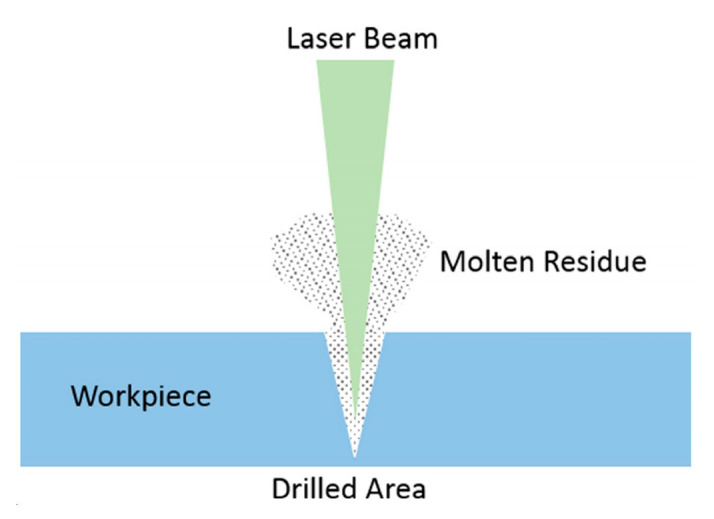
Laser drilling process. [Reproduced with permission from Rahim et al.; published by ASME, 2017] [176].

Laser processing is a non-contact method of removing material, which is also commonly referred to as laser drilling and laser cutting. Laser drilling involves the use of a focused pulse laser to deliver energy to a material, causing it to evaporate layer by layer until a hole is created [176]. In Figure 12, laser cutting is a technique that involves focusing laser light onto a material’s surface, resulting in an increase in temperature, softening, or even evaporation. The molten material is then removed using high-pressure gas that is coaxially blown off [177]. The quality of SiCp/Al processing is influenced by factors, such as laser power, processing conditions, and others. Several scholars have conducted relevant experimental research on the process.

During the drilling process, defects such as cracks and recast layers are typically caused due to the exceedingly high energy density of the laser. The quality of hole processing when using different types of laser drilling is a matter of concern, and an evaluation of laser drilling quality generally involves considering various factors, including hole depth, taper, and heat-affected zone (HAZ) [178]. In a study conducted by Padhee et al. [179], the laser drilling process of SiCp/Al materials was experimentally investigated using a YAG laser. The parameters of hole taper, splashing, and heat-affected zone were measured at various levels of three main influencing factors (pulse width, pulse number, and SiC particle concentration). A mathematical model was then developed using RSM to predict the response. The optimal values for minimizing parameters related to taper, splashing, and heat-affected zone were found to be a pulse width of 481.35 μs, a pulse frequency of 2.98 pulses per second, and a SiC particle mass percentage of 2.61.

Li et al. [180] utilized a high-power fiber laser to cut SiCp/Al composite materials that were 5.0 mm thick. They designed a full factorial experiment and determined that the significant factors influencing the incision width of the composite material were laser power, cutting speed, and auxiliary gas pressure. Additionally, they found that the significant influencing factors for the incision angle were cutting speed and auxiliary gas pressure. In both groups of experiments, the cutting speed was identified as the most significant factor, contributing 40% and 47%, respectively, according to the corresponding contribution rate (PCR). Furthermore, they discovered that at a cutting speed of 2500 mm/min, a laser power of 4000 W, and a gas pressure of 8 bar, they could achieve the minimum pit depth on the surface of the SiCp/Al composite. This finding supported the notion that a combination of high laser power and high cutting speed can lead to efficient material removal and improved processing quality. In a separate study, Zhang et al. [181] employed a high-power fiber laser system (IPGYLS-5000) to conduct experiments on laser cutting of SiCp/Al, using various process parameters. They analyzed the surface morphology and cross-section characteristics of the processed materials. They found that the threshold for successfully cutting 1.0 mm thick samples was 90 J/mm, while for cutting 5.0 mm thick samples, it was 450 J/mm.

**Figure 13:**
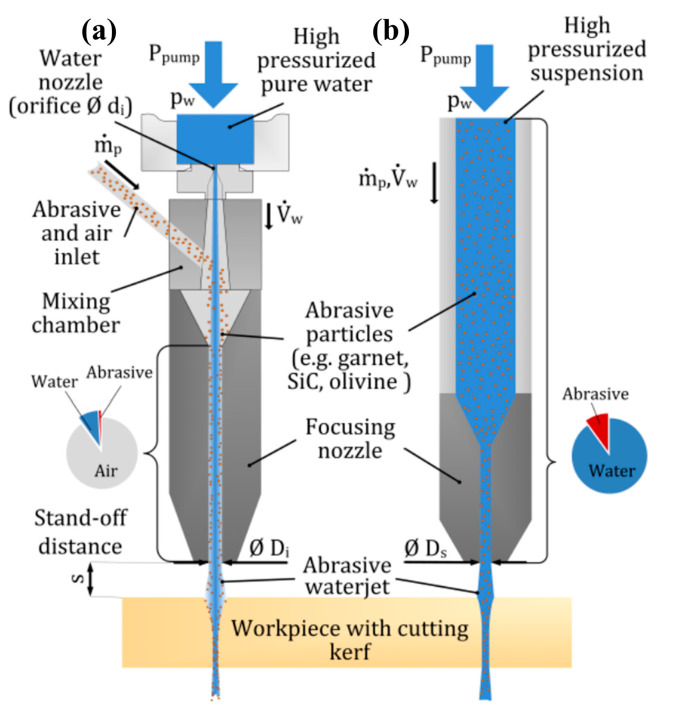
(**a**) Injection method and (**b**) suspension method. Ref. [184].

The abrasive water jet (AWJ) is a unique “cold” machining process that utilizes a high-pressure, high-flow rate water jet, as well as an abrasive slurry, to process target materials. It offers several advantages, such as no thermal deformation, low cutting force, and strong versatility [182]. The mechanism of the AWJ involves introducing abrasive particles, typically garnet, into a high-pressure, high-speed water flow. This allows the water’s momentum to transfer to the abrasive particles, resulting in the acceleration of numerous abrasive particles. Consequently, these particles mix with the water and form a high-speed jet that is directed towards the target material for erosion cutting [183]. The AWJ process can be divided into two types, depending on the method used for jet generation: the abrasive injection jet (three-phase—air, abrasive, water) and the abrasive suspension jet (two-phase—abrasive, water). The device schematic diagrams can be seen in Figure 13 [184]. However, due to its high operational difficulty, the suspended jet technology has yet to be widely implemented in the industry.

The main parameters that affect the quality of AWJ machining include water jet pressure, lateral displacement rate, spacing distance, abrasive type, abrasive size, etc. Scholars have conducted relevant process experiments to explore the AWJ processing of SiCp/Al materials.

Srinivas and Babu et al. [185] observed that the choice of abrasive, specifically the type of abrasive material and particle size, depends on the size of SiC particles in SiCp/Al material. They used 60-, 80-, and 120-mesh size garnet and SiC abrasive for AWJ machining of SiCp/Al material, respectively. The results showed that selecting 80-mesh SiC abrasive achieves a higher penetration depth of AWJ into SiCp/Al materials. They also noted that water jet pressure and transverse velocity are crucial factors in achieving higher penetration depth in trapezoidal SiCp/Al with a thickness of 70 mm. Furthermore, Srinivas and Babu et al. [186] conducted a study using SiCp/Al materials with volume fractions of 5%, 10%, 15%, and 20% to investigate the jet penetration ability of AWJ in SiCp/Al. They found that during the AWJ processing of SiCp/Al, water jet pressure and transverse velocity had the most significant impact on the cutting process. Additionally, as the hardness increased, the contribution of water jet pressure significantly decreased, while the contribution of the mass flow rate only slightly increased. These findings suggest that selecting a reasonable abrasive mass flow rate and transverse velocity is necessary when machining harder materials with AWJ to achieve higher penetration depth. Arul and Kumaresan et al. [187] designed orthogonal experiments to examine the effects of water jet pressure, lateral displacement rate, and spacing distance on the surface roughness of SiCp/Al, which are factors related to AWJ. They found that the lateral displacement rate had the most significant influence on SiCp/Al surface roughness during AWJ processing, followed by water jet pressure and spacing distance. The optimal combination of parameters for SiCp/Al surface roughness was determined to be a lateral displacement rate of 30 mm/min, water jet pressure of 90 MPa, and spacing distance of 2 mm. Similarly, Pruthviraju and Balamurugan et al. [188] conducted a similar investigation on the AWJ processing of composite materials. They identified water jet pressure, lateral displacement rate, and spacing distance as the main influencing factors and conducted orthogonal experiments. The research results revealed that the spacing distance and lateral displacement rate significantly affected the material removal rate, while water jet pressure and spacing distance significantly influenced the material surface roughness. Two sets of optimal experimental conditions were obtained, including the AWJ processing conditions for achieving the optimal material removal rate: lateral displacement rate of 29.2 mm/min, water jet pressure of 20 bar, and spacing distance of 1 mm. The AWJ processing conditions for obtaining the optimal material surface roughness included a lateral displacement rate of 21 mm/min, water jet pressure of 220 bar, and spacing distance of 1.1 mm.

Special processing offers certain advantages in SiCp/Al composite materials. Although it allows for the processing of relatively complex shapes, there is still a need to enhance production efficiency and processing accuracy.

## 8. Hybrid Machining of SiCp/Al

Traditional machining methods exhibit bottlenecks in improving machining performance, such as severe tool wear and poor surface integrity [189]. With the demand for high-dimensional accuracy, high efficiency, and low damage of composite materials, currently, the processing methods applied to hard and brittle materials mainly rely on ultrasonic-assisted processing technology [190]. These include ultrasonically assisted turning, ultrasonic-assisted milling [191,192,193], ultrasonic-assisted drilling, and ultrasonic-assisted grinding [194,195]. In this technology, additional excitation vibrations with ultrasonic frequency and amplitude are applied to the tool or workpiece. Its characteristic is that the tool or workpiece undergoes multiple separations and contacts during the machining process to change its material removal mechanism [196]. The UAM process has been proven to reduce cutting force and improve tool life when machining very hard and brittle materials [197].

**Figure 14:**
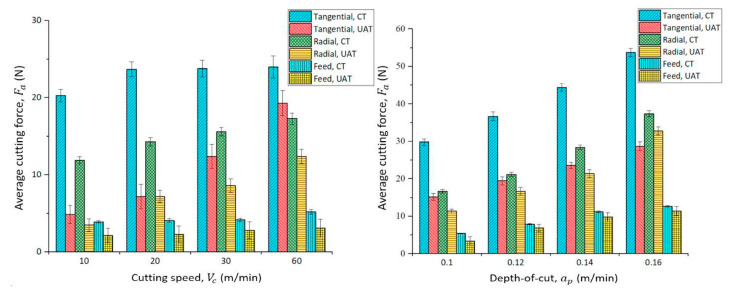
Average cutting force results [201].

In the process of ultrasonic vibration-assisted machining, ultrasonic vibration not only enhances the Al matrix and exhibits good resistance to adhesion but also induces the fracture of SiC particles [198]. This reduces the occurrence of SiC particles being pulled out from the surface and forming pits [199], as well as the occurrence of edge fracture [200], thereby improving the surface quality of the machined material. Kim et al. [201] conducted a series of comparative turning experiments using two sizes of SiC particle-reinforced aluminum matrix composites (217XG, 225XE) and concluded that compared to conventional turning (CT), ultrasonic vibration-assisted turning (UAT) resulted in decreases of 60.5% and 29.5% in the average tangential cutting force for 217XG and 225XE, respectively. Furthermore, the surface roughness increased by 33% and 52.5% for 217XG and 225XE, respectively, as shown in Figure 14. Gao et al. [202] verified that the ultrasonic vibration milling of SiCp/Al can improve surface integrity and tool life. The machined surface of ultrasonic vibration-assisted end grinding is characterized by peaks and troughs, with the surface finish being nearly isotropic [203]. Das believes that ultrasonic-assisted grinding (UAG) helps in removing material through ductile modes and achieving high surface quality. Both rotary ultrasonic end-face grinding experiments [204] and rotary ultrasonic side grinding experiments [205] have proven this. By using a diamond grinding tool with a smaller diameter and a relatively low-speed ratio, an optimal integrated surface can be obtained [206]. Additionally, in order to better compare the cutting forces generated by ultrasonic vibration-assisted machining technology with conventional machining processes, Zhou et al. [207] proposed a mechanical model for predicting the grinding forces of SiCp/Al composite materials in ultrasonic vibration-assisted grinding.

Ultrasonic vibration-assisted machining and conventional machining share similar principles in terms of process parameters [208]. Both techniques result in the PCD milling cutter’s side wear remaining linearly related to the milling length [209]. The difference in ultrasonic amplitude has an impact on the processing effects: as the amplitude increases, the chips gradually transition from curly to flaky. A low setting value for amplitude will not yield significant improvements in surface quality during machining. The minimum amplitude value is 5 μm [210]. When evaluating the surface quality of ultrasonic vibration-assisted machining, it is preferable to use three-dimensional surface dimensions (Ds) as they are more sensitive to defects on the surface of SiCp/Al composite materials compared to Sq. The order of influence of process parameters on surface roughness Sq is spindle speed, vibration amplitude, cutting depth, and feed speed. Conversely, the order of influence of process parameters on Ds is feed speed, spindle speed, vibration amplitude, and cutting depth [211].

The hybrid application of other processing technologies has effectively solved the challenge of processing SiCp/Al composite materials. For instance, a new method that combines ultrasonic vibration-assisted processing with laser heating-assisted processing has successfully adjusted both the ultrasonic amplitude and laser temperature. This simultaneous adjustment leads to a comprehensive reduction in force and thermal softening effect of the material, ultimately improving its processability and resulting in an ideal processing surface [212]. Additionally, this process unexpectedly enhances the precision of processing dimensions and suppresses both surface and subsurface damage [213].

The hybrid LUAM technology incorporates several technologies, including ultrasonic vibration-assisted laser turning (UVLAT), laser-induced oxidation-assisted micro-milling (LOM), laser ablation-assisted ultrasonic grinding (LAAUG), and ultrasonic-assisted laser hole drilling, among others.

The LUAM technology, which combines various techniques, effectively eliminates plasticity in the material by modifying the properties of the aluminum-based silicon carbide material. This modification ensures high quality on the processed surface. However, due to the material’s tendency to appear in the form of small particle fragmentation and particle pressing, the aluminum matrix is susceptible to developing cracks and pits when heated. This results in fewer surface defects and improved surface roughness. In addition, the chip surface shows smoother texture, increased thickness, and fewer cracks when observed [214]. Although the softening of the material extends the lifespan of the tool, the wear pattern and elemental composition of both the tool surface and processed surface remain consistent across different processes, with mechanical wear being the primary contributing factor [215]. Notably, micro chipping is noticeable on the front cutting edge, and shallow micro grooves are present along the cutting direction on the rear cutting edge [216]. Moreover, the cutting speed and laser power have no influence on the rate of tool wear [217]. Consequently, reducing tool wear in LUAM technology becomes even more challenging.

**Figure 15:**
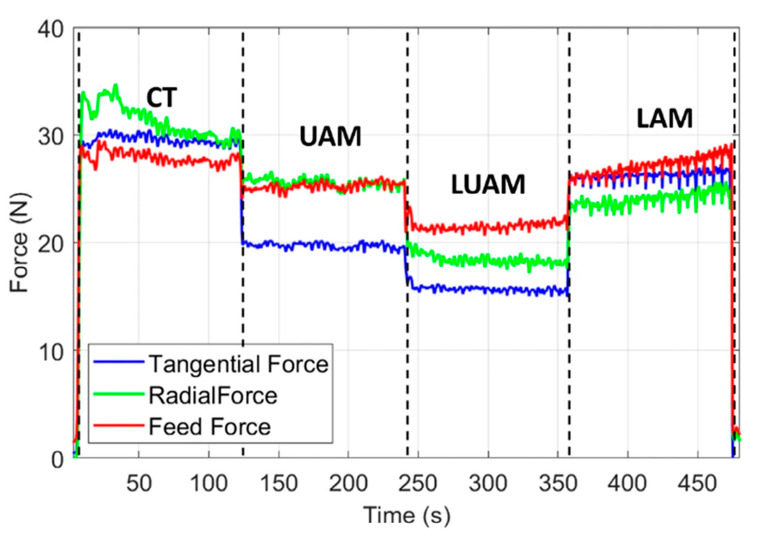
Comparison of cutting forces for four processes [218].

The ultrasonic vibration-assisted laser-assisted turning (UVLAT) technology has been proven to achieve the lowest cutting force for the tangential, radial, and feed force components. When compared to conventional turning (CT), ultrasonic-assisted turning (UAT), and laser-assisted turning (LAT), LUAM technology reduces the cutting forces in the tangential, radial, and feed directions by 70.1%, 59%, and 43%, respectively [218]. Figure 15 displays a chart comparing the cutting forces of these four processes in the tangential, radial, and feed directions. While the reduction in cutting forces is significant with the implementation of LUAM, it is important to carefully select the processing parameters, as achieving a lower surface quality requires thorough consideration [219]. The cutting force, radial force, and feed force all decrease with an increase in laser power. Similarly, an increase in laser power leads to a decrease in surface roughness [220]. However, it is crucial to avoid excessive amplitude of ultrasonic vibration and laser power, as these can cause severe deterioration of the surface quality [221].

The laser-induced oxidation-assisted micro-milling (LOM) processing method is not just practical but also efficient. This technique produces a porous and easily removable altered layer on the surface of composite materials, resulting in a significant reduction in the force exerted by particles on the tool. Consequently, tool wear is slowed down [222]. Additionally, the post-processed surface roughness is notably decreased, with a minimum Sa of approximately 100 nm [223]. Ultrasound longitudinal torque milling (ULTM) combines the advantages of ultrasound longitudinal milling and twist milling, allowing it to achieve high material removal rates and excellent surface quality. By setting the cutting-edge trajectory as a spiral and variably adjusting the spiral angle sinusoidally, the processed surface quality can be greatly improved [224].

Laser-assisted ablation-aided ultrasonic grinding (LAAUG) technology enables the achievement of heat-damage-free machining surfaces, effectively addressing the limitations of traditional grinding methods. Although the wear pattern of the grinding wheel remains consistent with conventional grinding, which includes grain displacement and fracture wear, the reduction in grinding force effectively suppresses the wear rate of the grinding wheel. Additionally, this process greatly enhances processing quality and efficiency [225].

The development of ultrasonic-assisted laser drilling technology not only increases hole depth but also effectively improves the hole wall quality of blind holes and through holes [226]. This improvement can be attributed to grain refinement and material strengthening in the process action area, which reduces the tool’s impact toughness. However, the selection of the pulse width is crucial in determining the degree of grain refinement of the material [227]. To further enhance drilling efficiency, femtosecond laser drilling technology has been introduced. However, this process can cause re-solidification and redeposition of particles on the hole wall. The use of ultrasonic vibration (US)-assisted laser drilling process can overcome this drawback and ensure the aspect ratio (depth to diameter ratio) and surface smoothness of the manufactured micro-holes [228].

By setting appropriate process parameters, such as amplitude, vibration direction, and laser power, the LUAM process can effectively address the challenges of precision machining for difficult-to-machine materials, making it a high-quality, high-efficiency, and precision-machining method [229].

In short, hybrid machining not only has unique advantages in processing high-volume-fraction SiCp/Al composite materials but can also reduce cutting force, slow down tool wear, minimize surface defects, and improve surface quality. Additionally, it can effectively solve the vibration problem when processing SiCp/Al thin-walled workpieces.

## 9. Conclusions

This article provides a comprehensive review on the machinability of SiCp/Al composite materials, focusing on various machining techniques, such as turning, milling, drilling, grinding, special machining, and composite machining. The priority is given to achieving high efficiency and quality in material processing. When selecting conventional processing methods (turning, milling, drilling, grinding) and their corresponding parameters (processing speed, cutting depth, and feed speed), it is crucial to align them with the requirements of composite material processing. Optimal processing conditions involve high speed, small depth of cut, and low feed rate, as they contribute to a better finish. However, material parameters like SiC particle content and size still play an important role in determining processing quality. Despite not being limited by material strength and hardness, special processing technologies, such as EDM, WEDM, CCEDM, PMEDM, ECJM, AWJ, and Jet ECM, have relatively low processing efficiency. To overcome the challenges in machining SiCp/Al composite materials, the use of assisted hybrid processing technologies has proven effective in achieving efficient and high-quality processing.

## Figures and Tables

**Table 1:** Performance parameters of aluminum matrix composite materials.

Material	Volume Fraction (%)	Density (g/cm^3^)	Elastic Modulus (GPa)	Strength (MPa)	Specific Stiffness E/p	Coefficient of Expansion (10^−6^/K)	Thermal Conductivity (W/m·K)
SiCp/Al	15	2.82	100 ± 5	560	35.5	17.0 ± 1	150 ± 5
	20	2.85	110 ± 5	580	38.6	16.0 ± 1	160 ± 5
	25	2.86	115 ± 5	600	40.2	14.3 ± 1	170 ± 5
	30	2.89	125 ± 5	560	43.3	13.0 ± 1	175 ± 5
	45	2.92	160 ± 5	500	54.9	11.5 ± 1	190 ± 5
	55	2.95	195 ± 5	450	66.1	9.5 ± 1	200 ± 5
	60	2.98	200 ± 5	420	67.1	8.3 ± 1	205 ± 5
	65	3.01	215 ± 5	400	71.4	7.5 ± 1	210 ± 10
	70	3.02	220 ± 5	380	72.8	7.0 ± 1	215 ± 10

**Table 2:** Recommended parameters for the milling of SiCp/Al composites.

Tool	Matrix	SiC (Volume Fraction)	Parameter	Remark
Carbide insert with a 0.8 mm uncoated tool nose radius [89]	Al7075 alloy	40%	Cutting speed 170 m/min, depth of cut 0.8 mm and a feed per tooth 0.08 mm/tooth.	Best surface quality
PCD tools a front angle of 0°, a back angle of 20°, and an edge radius of 0.01 mm [90]	Al2009	20%	Cutting speed of 47.10 m/min, feed rate of 10 mm/min, and depth of cut of 80 μm,	better surface roughness Ra0.029 μm.
A single flute Φ1 mm PCD end mill [91,92]	Al2024	45%	The suitable value of the feed rate is 30 mm/min.	best surface quality
End mill cutter (Φ16 mm) with 2 uncoated cemented carbide inserts [93]	A356 aluminum alloy	10%	Cutting speed 200 m/min, feed rate 0.1 mm/min, depth of cut 0.2 mm	The minimal surface roughness and cutting forces
CVD diamond-coated end mills [94]	Al7075	30%	Tool rotation speed of 6500 r/min, feed rate of 762 mm/min and depth of cut of 3 mm.	A high material removal rate (MRR) to tool wear and surface roughness ratio
PCD [95]	Al	65%	Tool rake angle 5, tool clearance angle 5, corner radius 0.4 mm, milling depth 50 mm, and milling speed 300 m/min.	Reduce surface machining defects
Three different cutting tools (uncoated, multi-layered, and nano TiAlN coated) [96]	123 L aluminum alloy	10% SiC under 32 μm	Uncoated tool: cutting speed 60 m/min, feed rate 0.04 mm/r; multi-layered tool: cutting speed 78 m/min, feed rate 0.12 mm/r	Multi-layered tool 0.302 μm
PCD end mill [97]	Al2024	60%	Spindle speed 14,000 r/ min, feed rate 6 mm/min, and milling depth 40 μm.	surface roughness 0.238 μm.

**Table 3:** Summary of research work.

Author	Material	Parameters	Remarks
Shu-Tao Huang [103]	Al- SiCp (65% vol)	Cutting speed and feed rate	Abrasive wear and adhesive wear of the flank face.
Gul Tosun & Mehtap Muratoglu [114]	Al 2124- SiCp (17% vol)	Types of drills, Point angle, Aging	As the point angles of HSS and TiN-coated HSS drills increase, the damage zone increased. But for solid carbide drills, the damage zone decreased. Peak-aged materials give the best performance
Ferit Ficici [115]	Al 7075-SiCp (5%, 10%, 15% vol)	Types of the drill, materials parameters	The surface roughness values increased with increasing the feed rate and decreased with increasing the cutting velocity
Gul Tosun & Mehtap Muratoglu [116]	Al 2124- SiCp (17% vol)	Dry drilling tests, spindle-speed, feed rates, drills, point angles, heat treatment	Surface roughness decrease with the increasing feed rate Hard-carbide tools produce a better surface finish Increasing the point angles the surface roughness decreased.
Chang Liu [117]	Al6061-SiCp (15%, 30%, 45% vol)	Dry drilling tests, experimental parameters	Pvf contributes 44.1%, 74.1%, and 74.2% on cutting force, burr height, and surface roughness
Li Zhou [118]	Al-SiCp (56%)	Electroplating diamond drills, cutting speed	The average surface roughness increased with the increasing drilling speed, and the surface roughness in dry conditions is higher than that in wet conditions.

## Data Availability

All data used in this study are available upon request from the corresponding author.
